# Epidemiology and clinical characteristics of acute respiratory tract infections among hospitalized infants and young children in Chengdu, West China, 2009–2014

**DOI:** 10.1186/s12887-018-1203-y

**Published:** 2018-07-05

**Authors:** Jiayi Chen, Pengwei Hu, Tao Zhou, Tianli Zheng, Lingxu Zhou, Chunping Jiang, Xiaofang Pei

**Affiliations:** 1 Department of Public Health Laboratory Sciences, West China School of Public Health (No.4 West China Teaching Hospital), Sichuan University, 16#, Section 3, Renmin Road South, Chengdu, 610041 Sichuan People’s Republic of China; 2 Research Center for Occupational Respiratory Diseases, West China School of Public Health (No.4 West China Teaching Hospital), Sichuan University, 16#, Section 3, Renmin Road South, Chengdu, 610041 Sichuan China; 3 Shenzhen Nanshan Center for Disease Control and Prevention, 95#, Nanshang Road, Shenzhen, 518054 Guangdong China; 4 Chongqing Yuzhong District Center for Disease Control and Prevention, 254#, Heping Road, Yuzhong District, Chongqing, 400010 China

**Keywords:** Acute respiratory infections, Epidemiology, Clinical characteristics, Pediatrics, West China

## Abstract

**Background:**

Acute respiratory infection (ARI) is the leading cause of morbidity and mortality in pediatric patients worldwide and imposes an intense pressure on health care facilities. Data on the epidemiology profiles of ARIs are scarce in the western and rural areas of China. The purpose of the current study is to provide data on the presence of potential pathogens of ARIs in hospitalized children in Chengdu, west China.

**Methods:**

Respiratory specimens were obtained from hospitalized patients (under 6 years old) with ARIs in a local hospital in Chengdu. Eight respiratory viruses were identified by PCR and 6 respiratory bacteria by biochemical reactions and Analytical Profile Index (API). Pathogens profiles, clinical characteristics and seasonality were analyzed.

**Results:**

Fifty-one percent of patients were identified with at least one respiratory pathogen. Human rhinovirus (HRV) (23%), Respiratory syncytial virus (RSV) (22.7%) was the most commonly identified viruses, with *Klebsiella pneumoniae* (11.5%) the most commonly identified bacterium in the study. The presences of more than one pathogen were found, and multiple viral, bacterial, viral/bacterial combinations were identified in 14.9, 3.3 and 13.9% of patients respectively. Respiratory viruses were identified throughout the year with a seasonal peak in December–February. Pathogens profiles and clinical associations were different between infants (< 1 year of age) and older children (> 1 year of age). Infants with ARIs were more likely to have one or more viruses than older children. Infants identified with multiple pathogens had significantly higher proportions of tachypnea than infants that were not.

**Conclusions:**

This study demonstrated that viral agents were frequently found in hospitalized children with ARI in Chengdu during the study period. This study gives us better information on the pathogen profiles, clinical associations, co-infection combinations and seasonal features of ARIs in hospitalized children, which is important for diagnoses and treatment of ARIs, as well as implementation of vaccines in this area. Moreover, future efforts in reducing the impact of ARIs will depend on programs in which available vaccines, especially vaccines on RSV, HRV and *S. pneumoniae* could be employed in this region and new vaccines could be developed against common pathogens.

## Background

Acute respiratory infections (ARIs) remain one of the most common major public health threats [1]. There were approximate 11.9 million episodes of severe acute lower respiratory infections (ALRI) resulted in hospital admissions in young children worldwide [2], and ARIs-related pneumonia was one of the leading cause of death that due to infectious disease in China (> 30,000 deaths annually) as well as globally (935,000 in 2013) [3, 4]. There were, as have been suggested, associations of several viral agents with ARIs, such as: respiratory syncytial virus (RSV), human rhinovirus (HRV), human metapneumovirus (HMPV), influenza virus (IFV), parainfluenza virus (PIV), adenovirus (ADV) and human bocavirus (BoV), accounting for about 35–87% of children with ARI [5]. Viral co-infections occurred in 4–33% of children hospitalized with ARIs, and may indicate an increasing risk for clinical outcome [6, 7]. Further, bacterial infections such as: *Streptococcus pneumoniae*, *Haemophilus influenzae*, *Staphylococcus aureus*, *Pseudomonas aeruginosa* and *Klebsiella pneumoniae*, et al., were commonly observed in the later stage of diseases due to immune-compromised viral infections [8].

Similarities among ARI symptoms hampers the diagnostic and therapeutic efficacy among infected children, and inappropriate medication options may lead to the potential of viral escape mutants or bacterial resistance [8]. The composition of ARIs is geographically diverse and is largely associated with the epidemic status of each ARIs and climate conditions [9, 10]. The prevalence of ARIs varies from 14.6 to 94.3% among hospitalized children with respiratory infections in some metropolitan cities such as Beijing, Shanghai and Shenzhen in China [11–14]. However, in the underdeveloped interior areas of China, epidemiology profiles of ARIs are seldom reported. Chengdu is a mega-city in the southwest of China and has a population of more than 15 million people. Flu vaccination rates were pretty low in this area, which were 2.18, 1.69, 1.82 and 1.63% respectively from 2010 to 2013 [15]. The purpose of the current study is to investigate the profiles of respiratory pathogens and epidemiology characteristics of ARIs in hospitalized children in Chengdu. The results were expected to help improve diagnosis and optimization of therapeutic regimens of ARIs in this area.

## Methods

### Study design and patient population

The cross-sectional study was conducted monthly at a sentinel tertiary women’s and children’s hospitals in Chengdu, West China, from September 2009 to February 2014. A total of 1992 hospitalized children younger than 6 years old that presented symptoms of ARIs were recruited. Inclusion criteria for cases were (i) acute infection, for example fever, WBC anomaly, shivering; (ii) respiratory symptoms such as cough, rhinorrhea, pharyngalgia, expectoration, nose/throat congestion, shortness of breath, abnormal breathing sounds or dyspnea. The study protocol was approved by the Medical Ethics Committee of Sichuan University and written informed consents were obtained from the parents or the caregiver before collecting samples.

Samples including nasopharyngeal aspirates, sputum, throat swabs, blood and bronchoalevlar lavage fluid were collected by qualified medical personnel. The demographic information and medical records of the participated patients were also collected. All samples were delivered to the Microbiology Laboratory of Department of Public Health Laboratory Sciences, Sichuan University immediately after collection samples via cold chain transportation and stored at − 80 °C.

### Pathogen analysis

Nasopharyngeal aspirates, sputum, throat swabs and bronchoalevlar lavage fluid were used for respiratory virus analyses. Viral RNA and DNA were extracted by Viral Nucleic Acid Extraction Kit (Geneaid, Taiwan District) according to the manufacturer’s instructions. cDNA were synthesized with reverse transcription kit (BIO-RAD, California, US). Primers and multiplex PCR conditions for IFV [16], RSV [16], PIV [17], ADV [18], HMPV [13], human coronavirus (HCoV) [19], HBoV [20] and HRV [17] have been described previously. Primers were synthesized by Life Technology Corp. (Shanghai, China) and PCRs were performed using PCR Mastermix (Tiangen Company, China) and the S1000™ Thermal Cycler (BIO-RAD, California, US). Respiratory viruses were initially identified by the size of PCR products following agarose gel electrophoresis, with confirmation by DNA sequencing (Life Technology Corp., Shanghai, China).

Nasopharyngeal aspirates, sputum, blood and bronchoalevlar lavage fluid were used for bacterial analyses. For isolation of bacteria, specimens were cultured on sheep blood agar, chocolate agar and MacConkey’s agar plates. Isolated bacteria were primarily evaluated by colonial morphology, Gram staining, and were finally identified by biochemical reactions and API system. *S. pneumoniae* were differentiated by Optochin sensitivity and *Group A streptococcus* were identified by bacitracin sensitivity. *P. aeruginosa* and *K. pneumoniae* were identified by API 20E. *H. influenzae*, *S. aureus* were differentiated by API HN, API Staph respectively.

### Statistical analysis

The collected data were analyzed through SPSS version 19.0. Descriptive statistics were done in the form of means, frequencies and ranges of the variables. Continuous variables were expressed as means with their standard deviations. Categorical variables such as age groups and their associations with proportions of certain pathogens were analyzed using the chi-square test or the Fisher’s exact test. Significant differences, associations and interrelationships of the variables were assessed at a level of *P* < 0.05.

## Results

### Profile of enrolled patients

The median age of the patients was 9 months (ranged from 1 days to 6 years old), with 54% of patients under 1 year of age. Of 1992 enrolled children, 1185 were boys and 807 were girls (gender ratio of 1.47: 1). Cough was found in 1354 (68.0%) of the children, followed by fever (51.9%, 1033) and expectoration (29.0%, 578). The median number of days between symptom onset and hospitalization was 10 days. 52.5% (1045/1992) of the patients were diagnosed with pneumonia by chest X-ray. Among all the cases, 9 children died during the study period, including 8 boys and 1 girl.

### Results of pathogen analysis

Respiratory viruses were analyzed in 1764 samples. HRV testing was added in 2012 and HRV was analyzed in 795 samples. One or more respiratory viruses were identified in 51.0% of the patients (Table 1). HRV (23.0%, 183/795) and RSV (22.7%, 401/1764) were the most commonly identified viruses in hospitalized children, followed by PIV (13.4%, 236/1764), HBoV (8.4%, 149/1764) and ADV (6.2%, 110/1764). Other viruses such as IFV (4.4%), HMPV (2.2%) and HCoV (0.6%) were identified in small proportions. Multiple viral combinations in samples were found, including 223 dual, 37 triple and 3 quadruple combinations. Common combinations are listed in Table 2.

**Table 1:** Pathogen profiles and distributions of viral agents among age groups

	< 6 months, *n* = 799		6 months-1 year, *n* = 193		1-3 years, *n* = 351		> 3 years, *n* = 421		All ages, *n* = 1764	
	No.%	Mixed	No.%	Mixed	No.%	Mixed	No.%	Mixed	No.%	Mixed
Positive	414 (51.8)^a^		130 (67.4)		194 (55.3)		162 (38.5)		900 (51.0)^a^	
Single	297 (33.0)^b^		92 (10.2)		127 (14.1)		121 (13.4)		637 (70.8)^b^	
Mixed	117 (13.0)^c^		38 (4.2)		68 (7.6)		40 (4.4)		263 (29.2)^b^	
IFV	29 (3.2)^b^	21 (72.4)^c^	11 (1.2)	8 (72.7)	18 (23.1)	15 (83.3)	20 (25.6)	8 (40.0)	78 (4.4)^a^	52 (66.7)
RSV	212 (23.6)	69 (32.5)	61 (6.8)	27 (44.3)	82 (9.1)	29 (35.4)	46 (5.1)	24 (52.2)	401 (22.7)	149 (37.2)
PIV	91 (10.1)	47 (51.6)	42 (4.7)	23 (48.9)	61 (6.8)	30 (49.2)	42 (4.4)	16 (38.1)	236 (13.4)	116 (49.2)
ADV	29 (3.2)	22 (75.9)	25 (2.8)	10 (40.0)	33 (3.7)	20 (60.6)	23 (2.6)	9 (39.1)	110 (6.2)	61 (55.5)
HPMV	20 (2.2)	10 (50.0)	4 (0.4)	3 (75.0)	8 (0.9)	7 (87.5)	6 (0.7)	1 (16.7)	38 (2.2)	21 (55.3)
CoV	7 (0.8)	6 (85.7)	1 (0.1)	0 (0.0)	1 (0.1)	1 (100.0)	2 (0.2)	1 (50.0)	11 (0.6)	8 (72.7)
HBoV	62 (6.9)	26 (41.9)	19 (2.1)	10 (52.6)	44 (4.9)	23 (52.3)	24 (2.7)	13 (54.2)	149 (8.4)	72 (48.3)
HRV	95 (20.4)^d^	47 (49.4)	17 (3.7)	7 (41.2)	27 (5.8)	22 (81.5)	44 (9.5)	14 (31.8)	183 (23.0)^e^	90 (49.2)

^a^Case number and percentage by age group

^b^Case number and percentage among all positive cases

^c^Co-infection cases of each virus detected and percentages of number of positive cases

^d^Positive cases in the group that were analyzed for HRV was 465

^e^HRV was analyzed in 795 samples (HRV testing was added in 2012)

**Table 2:** Common combinations of analyzed pathogens

	No.
Virus Combinations	
RSV + PIV	40
RSV + ADV	25
RSV + HRV	23
PIV + HBoV	18
HBoV+HRV	14
RSV + PIV + HRV	9
Virus/Bacteria Combinations	
RSV + *S. pneumoniae*	22
RSV + *K. pneumoniae*	16
IFV + *S. pneumoniae*	12
HRV+ *S. pneumoniae*	11
HRV+ *K. pneumoniae*	10

Six respiratory bacteria were analyzed among 1816 samples. One or more respiratory bacteria were identified in 26.2% (475/1816) of the patients. The most commonly detected bacteria were *K. pneumoniae* (11.5%, 209/1816), *S. pneumoniae* (9.5%, 173/1816) and *S. aureus* (4.0%, 73/1816), followed by *H. influenzae* (2.5%), *P. aeruginosa* (1.9%), and *Group A streptococcus* (0.4%). Multiple bacterial combinations in samples were found, including 52 dual and 8 triple bacterial combinations. *K. pneumoniae*/*S. pneumoniae* co-infection was the most frequent combination. Eight viruses and 6 bacteria were tested in 1728 samples, among which 883 (51.1%) were identified with at least one respiratory pathogen. 240 (27.2%, 240/883) were viral/bacterial combination among positive cases.

Among 9 death cases, 6 patients were identified with at least 1 respiratory pathogen, including 2 boys with single virus (IFV, HRV respectively), 2 boys with dual virus (PIV/HRV, PIV/HBoV respectively), 1 boy with triple virus (RSV/PIV/HRV) and 1 girl with both virus and bacteria (PIV/*S.pneumoniae*). None of the pathogens was identified in the rest of 3 boys.

### Respiratory pathogens and clinical characteristics

A univariate analysis was conducted to find associations between demographic and clinical characteristics with infection (Table 3). Infants tended to have a higher proportion of virus infection than older children (*P* < 0.001), with those from 6 to 12 months of age having the highest proportion (67.4%) (*P* < 0.001). Infants identified with viruses had higher proportion of fever (*P* < 0.001), cough (*P* < 0.001), runny nose (*P* = 0.007), expectoration (*P* < 0.001) and diarrhea (*P* = 0.031) than infants that were not. Older children with viral infections had a higher proportion of tachypnea (*P* = 0.007) than younger children (less than 1 year of age). In the virus-positive group, infants with viral co-infections had a significantly higher proportion of tachypnea (*P* = 0.033) than that with single viral infections.

**Table 3:** Clinical characteristics in infants with viral or bacterial workups

Clinical characteristics	Any Virus *n* = 544	No Virus *n* = 448	*P* Value	Any Bacteria *n* = 241	No Bacteria *n* = 773	*P* Value
Fever (> 37.5 °C)	208 (38.2)	108 (24.1)	0.000**	85 (35.3)	239 (30.9)	0.235
Cough	366 (67.3)	177 (39.5)	0.000**	171 (71.0)	379 (49.0)	0.000**
Runny nose	71 (13.1)	34 (7.6)	0.007**	28 (11.6)	76 (9.8)	0.465
Expectoration	146 (26.8)	66 (14.7)	0.000**	63 (26.1)	154 (19.9)	0.048*
Tachypnea	95 (17.5)	66 (14.7)	0.261	35 (14.5)	126 (16.3)	0.546
Dyspnea	93 (17.1)	94 (21.0)	0.122	47 (19.5)	145 (18.8)	0.851
Diarrhea	70 (12.9)	38 (8.5)	0.031*	27 (11.2)	85 (11.0)	1.000
Pneumonia	279 (51.3)	211 (47.1)	0.189	143 (59.3)	361 (46.7)	0.001**

* *P* < 0.05, ** *P* < 0.01

Demographic and clinical features for each analyzed respiratory virus were also examined. PIV and ADV were usually recognized in children aged from 6 to 12 months, whereas HBoV was mostly found in 1 to 3 years group, while HRV was found in all age groups (Table 1). PIV was associated with fever, cough and expectoration while HRV with cough, expectoration and diarrhea (Data not shown).

Among children tested for respiratory bacteria, older children had higher proportion of bacterial infections than infants (*P* = 0.010). *K. pneumoniae* was usually detected in infants and *S. pneumoniae*, *H. influenzae* were more common in older children. Infants with bacterial infections had higher proportion of cough (*P* < 0.001) and expectoration (*P* = 0.048).

Among 1728 children (with complete viral and bacterial workups), infants identified with any pathogen (Table 4) had higher proportions of fever (*P* < 0.001), cough (*P* < 0.001), runny nose (*P* = 0.011), expectoration (*P* < 0.001) and diarrhea (*P* = 0.009) than infants that were not. Pathogen-positive older children had a higher proportion of chest pain (*P* = 0.037). In the pathogen positive group, infants with viral/bacterial co-infection had significantly higher proportions of cough (*P* = 0.007) and tachypnea (*P* = 0.025).

**Table 4:** Clinical characteristics in infants with complete viral and bacterial workups

Clinical characteristics	Any Pathogen *n* = 332	No Pathogen *n* = 653	*P* Value	Virus Alone *n* = 412	Bacteria Alone *n* = 113	Virus/Baccteria Co-infection *n* = 128	*P* Value
Fever (> 37.5 °C)	69 (20.8)	243 (37.2)	0.000**	158 (38.3)	37 (32.7)	48 (37.5)	0.559
Cough	105 (31.6)	433 (66.3)	0.000**	262 (63.6)	71 (62.8)	100 (78.1)	0.007**
Runny nose	23 (6.9)	80 (12.3)	0.011*	52 (12.6)	10 (8.8)	18 (14.1)	0.455
Expectoration	44 (13.3)	166 (25.4)	0.000**	103 (25.0)	22 (19.5)	41 (32.0)	0.077
Tachypnea	57 (17.2)	104 (15.9)	0.649	69 (16.7)	9 (8.0)	26 (20.3)	0.025*
Dyspnea	69 (20.8)	117 (17.9)	0.301	70 (17.0)	25 (22.1)	22 (17.2)	0.445
Diarrhea	24 (7.2)	83 (12.7)	0.009**	56 (13.6)	13 (11.5)	14 (10.9)	0.680
Pneumonia	341 (52.2)	146 (44.0)	0.014*	198 (48.1)	64 (56.6)	79 (61.7)	0.002**

* *P* < 0.05, ** *P* < 0.01

Considering diagnoses and etiology, pneumonia was taken into consideration. Older children had significantly higher proportion of pneumonia (*P* < 0.001) than infants, with the highest rate observed in children 1 to 3 years of age. Infants identified with bacterial had a higher proportion of pneumonia than older children, and *S. aureus* (*P* = 0.004) and *S. pneumonia* (*P* = 0.009) were associated with pneumonia.

### Seasonal distributions

**Fig. 1:**
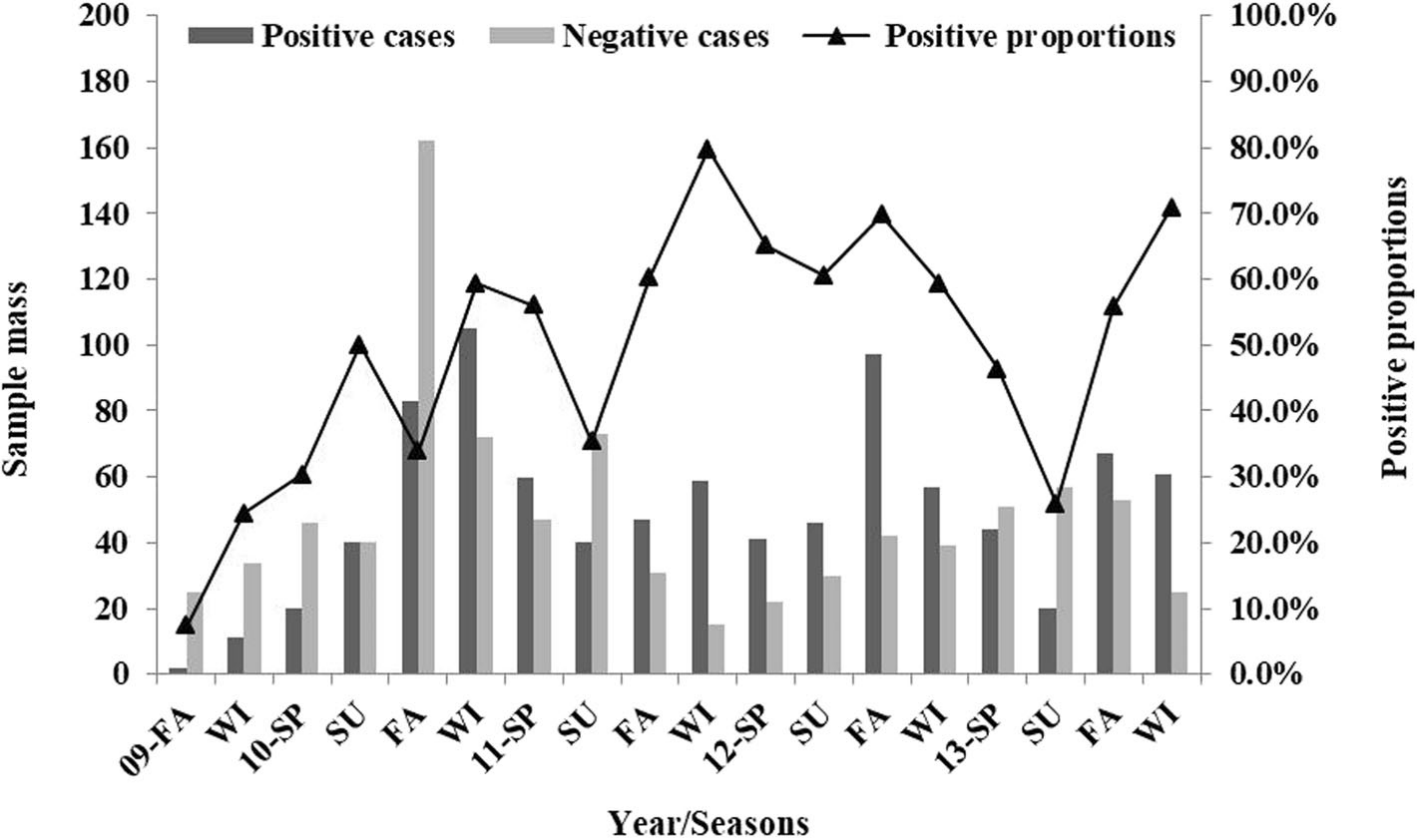
Seasonal distribution of ARIs in hospitalized children, Nov 2009 - Feb 2014. Spring (SP); Summer (SU); Fall (FA); Winter (WI)

**Fig. 2:**
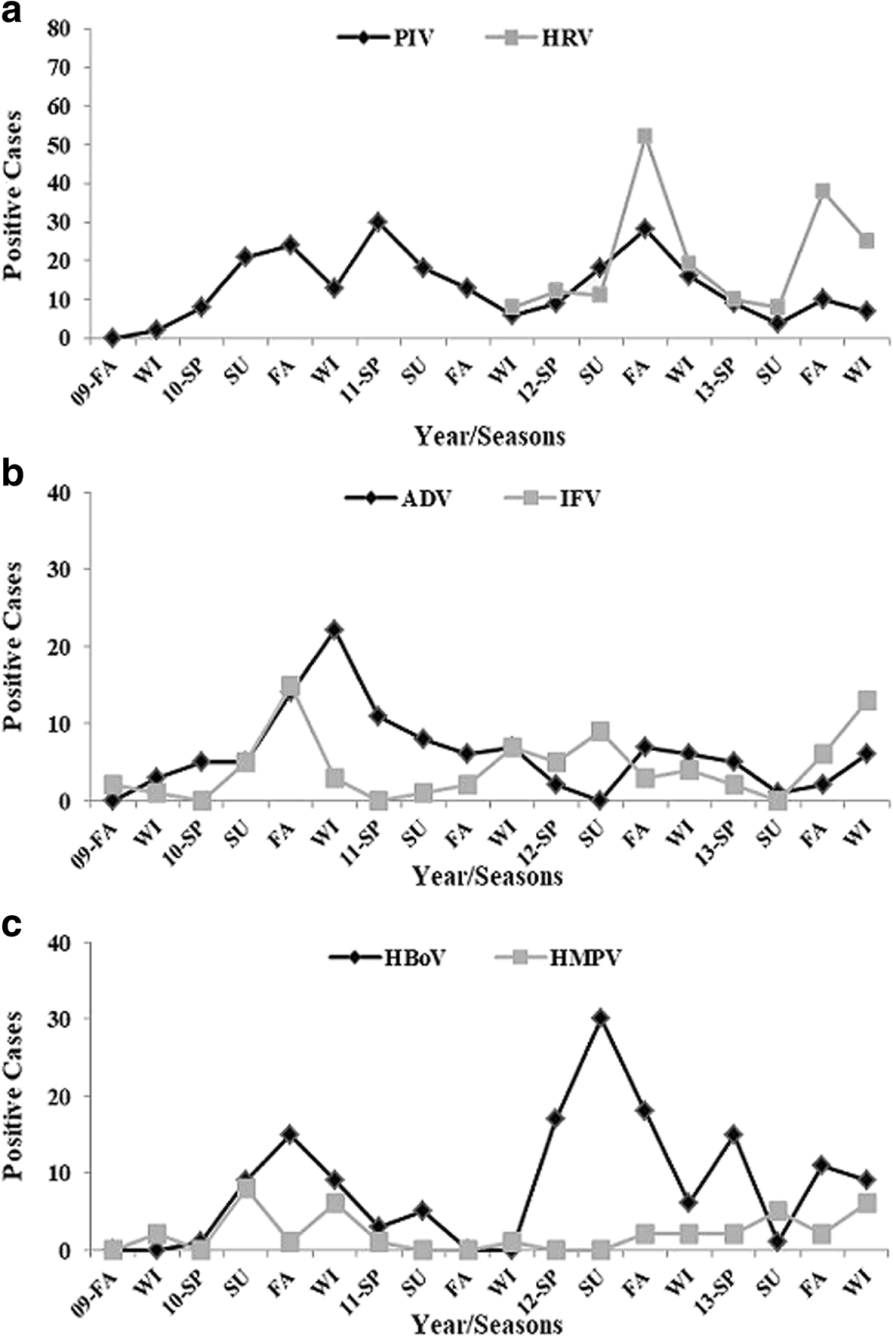
Seasonal distributions of respiratory viruses in hospitalized children, Nov 2009- Feb 2014. Spring (SP); Summer (SU); Fall (FA); Winter (WI)

Seasonal distributions of respiratory pathogens were estimated by admission dates for the included children. The seasonal detection rates ranged from 7.4 to 79.7% with a mean rate of 49.5%. Overall, viral infections were more likely to occur in winter months (December to February) with a decline in summer months (June to August) (Fig. 1). The same distribution was observed for ADV (Fig. 2). PIV and HRV were detected all year, with their detection peaks often in autumn. HBoV infections showed a peak in the summer season of 2010 and 2012. No regular seasonal variations with IFV and HPMV were observed during the study period.

## Discussion

To the best of our knowledge, this study is the first study to investigate the role of 8 respiratory viruses and 6 respiratory bacteria in ARIs among hospitalized children in Chengdu district, west China over a 5-year period. Therefore, these findings may be useful nationally and internationally equally.

The results confirmed a frequent viral etiology among 51% of children aged < 6 years presenting ARIs. A review of studies on positive proportions of respiratory viruses among patients with ARIs in different parts of China, such as Beijing [14] Shanghai [11], Shenzhen [12], Wuhan [21], Lanzhou [22], indicated high variability. Different incidences may be attributed to different age groups, samples collected, inclusion criteria, diagnostic methods, target pathogens and seasonality.

IFV, RSV, HPMV and HRV were the most commonly detected viruses in most regions. HRV and RSV were equally predominant in our study period. RSV is a major respiratory virus that causes respiratory illness including bronchiolitis, pneumonia, and wheezing [23]. RSV may bring about annual epidemics worldwide because of virus variability [24]. It is reported that RSV epidemic was associated with the alternate circulation of multiple genotypes and with the change of G protein [25]. HRV are usually associated with upper respiratory tract infections and are responsible for one half of all “common colds” [26]. The implementation of molecular methods has revealed HRV as an etiologic agent in lower respiratory tract infections (LRTI), associated with recurrent wheezing and asthma in infancy [26, 27].

Clinical presentations of respiratory infections may be overlapping and could not discriminate between respiratory viruses. However, infants identified with respiratory virus tended to have more severe symptoms, particularly those in the 7–12 months of age group. Age and exposure were two crucial factors for infection [8]. Immune status of infants is different from adults, and the amount of maternal antibodies attenuate distinctly, which would make infants susceptible to respiratory viral infections. The onset of disease is an interplay of immune pathology and viral pathology and prevention measures need to be taken into account [28].

It has been suggested that multiplex PCR techniques demonstrate a high detection rate of viral co-infections [29]. In this target population, mixed respiratory virus had a proportion of 14.9%, with RSV/PIV the most frequent combination. Previous research showed proportions of viral co-infection ranged from 4.0 to 24.7%, with RSV, IFV, HPMV the common viruses present in co-infections. Different incidence of co-infection may be due to high single infection rates of certain viruses, overlapping epidemic seasons, target pathogens as well as methodology. Evidence for increased clinical severity of viral single infections versus co-infections is controversial [30–33]. The impact of co-infection on clinical presentations may rely on the specific agent involved, as well as viral load [32, 33]. Experimental studies of simultaneous respiratory infections are scarce. However, one study showed by mathematical modeling, found that one virus can block replication of another due to competition for resources, which may have implications for the treatment regimens of simultaneous viral infections [34].

Despite vaccination, pneumonia still remains a serious public health issue in the world [35]. In this study, children > 1 year seemed to suffer more from bacterial infections than younger children. The WHO reports that pneumonia is the forgotten killer of children and one of the main disease burdens worldwide [36]. Pneumonia is the most serious result of ARIs and is often due to bacterial infection [37]. *S. pneumoniae, H. influenzae type b* are the leading causes of bacterial pneumonia in children worldwide [38, 39]. Under-nutrition, lack of breast-feeding, crowding, exposure to indoor air pollution, low birth weight and diarrhea have been identified as risk factors for pneumonia [40, 41]. Etiologic studies could provide information on the prevalence of bacterial infections, which may help achieve a reduction in child mortality.

The incidence of respiratory viral/bacterial co-infection in young children ranged from 1 to 44% [42]. In the current study, we observed a respiratory viral/bacterial co-infection proportion of 13.9% (240/1728). *S.pneumoniae, S. aureus, H. influenzae* and *Pseudomonas* species were common bacterial co-pathogens. Influenza pandemics over the last 100 years have strengthened the association of bacterial super-infection and influenza infection [42, 43]. There are mounting data indicating that virus infection can predispose to bacterial colonization and overgrowth, which adversely affect the pathogenesis of respiratory infections [44, 45]. Disruption of the epithelial barrier, up regulation of adhesion proteins, production of viral factors and alterations in immune responses are several known mechanisms [44, 45]. Increased morbidity with bacterial co-infections was found in children, leading to increased duration of mechanical ventilation, longer hospital stays and admission to pediatric intensive care units [42, 46]. However, the statistical association was weak and sporadic in some studies, and additional longitudinal cohort studies may be needed to reveal the contribution of bacterial to viral ARIs.

There was a paucity of study that was conducted for etiologies of ARIs among hospitalized children in this area. Including 14 different respiratory pathogens (both viruses and bacteria), recruiting almost 2000 hospitalized children, over 5-year period, this current study revealed pathogens profiles, co-infection pattern, clinical features, and seasonality among hospitalized children with ARIs in Chengdu, west China. There are however some limitations in this study. Outpatients were not recruited and the study lacked an asymptomatic control group. Positive rates may be somewhat lower as other potential viral pathogens were not included (e.g. enteroviruses, HCoV NL63 and HKU1). In addition, virus genotyping and strain identification (such as HRV A/B/C) would be helpful for understanding viral epidemics and associated disease severity.

## Conclusions

In conclusion, this 5-year consecutive surveillance research confirmed that respiratory viruses, especially RSV and HRV, were the leading potential cause of ARIs in hospitalized children in Chengdu, west China. Co-infections were associated with severity of illness in infants, who tended to have increased risks of ARIs. This study gives us better information on the pathogen profiles, clinical association, co-infection combinations, and seasonal features of ARIs in hospitalized children in this area. Moreover, future efforts in reducing the impact of ARIs will depend on a commitment to fund and implement programs to utilize available vaccines, especially vaccines on RSV, HRV and *S. pneumoniae* in in this region, and to develop new vaccines against common pathogens.

## Abbreviations

ADV: Human adenovirus
ARI: Acute respiratory infection
HBoV: Human bocavirus
HCoV: Human coronavirus
HMPV: Human metapneumovirus
HRV: Human rhinovirus
IFV: Influenza virus
LRTI: Lower respiratory tract infection
PCR: Polymerase chain reaction
PIV: Parainfluenza virus
RSV: Respiratory syncytial virus
